# Urban-rural disparities in the healthy ageing trajectory in China: a population-based study

**DOI:** 10.1186/s12889-022-13757-x

**Published:** 2022-07-23

**Authors:** Haomiao Li, Yixin Zeng, Li Gan, Yusupujiang Tuersun, Jiao Yang, Jing Liu, Jiangyun Chen

**Affiliations:** 1 School of Political Science and Public Administration, Wuhan University, Bayi Road No.299, Wuhan, Hubei Province China; 2 School of Health Management, Southern Medical University, No.1023-1063 Shatai Road, Baiyun District, Guangzhou, Guangdong Province China; 3 Guangzhou First People’s Hospital, Guangzhou, Guangdong Province China; 4 Institute of Health Management, Southern Medical University, No.1023-1063 Shatai Road, Baiyun District, Guangzhou, Guangdong Province China

**Keywords:** Health ageing, Urban and rural disparity, China, trajectory

## Abstract

**Purpose:**

The aim of this study is to measure the trajectory of healthy ageing among Chinese middle-aged and older population, and explore the disparity of the trajectory, as well as contributing factors, between urban and rural areas in China.

**Methods:**

A total of 9402 respondents aged 45 years and older interviewed in four waves (2011, 2013, 2015 and 2018) were selected from the China Health and Retirement Longitudinal Study. Healthy ageing score was calculated through item response theory. A latent growth mixture model (LGMM) was applied to distinguish the trajectory of healthy aging. A multinomial logistics regression model (MLRM) was used to explore the relationship between urban-rural areas and healthy aging trajectories, and further to explore associated factors in rural and urban areas separately.

**Results:**

The healthy ageing score was lower in rural areas than urban areas in each survey wave. Five classes (“continuing-low”, “continuing-middle”, “continuing-middle-to-high”, “significantly-declining”, “continuing-high”) were grouped through LGMM. The MLRM results showed that urban living was significantly associated with a higher likelihood of being healthy (for [continuing-low/continuing-high]: β = − 1.17, RRR = 0.31, *P* < 0.001, 95% CI = 0.18–0.53; and for [continuing-middle/continuing-high]: β = − 0.53, RRR = 0.59, *P* < 0.001, 95% CI = 0.49–0.71).

**Conclusion:**

Healthy ageing is a prominent objective in the development of a country, and rural-urban disparities are an essential obstacle to overcome, with the rural population more likely to develop a low level of healthy ageing trajectory. Prevention and standardized management of chronic diseases should be enhanced, and social participation should be encouraged to promote healthy ageing. The policy inclination and resource investment should be enhanced to reduce disparity in healthy ageing between urban and rural areas in China.

**Supplementary Information:**

The online version contains supplementary material available at 10.1186/s12889-022-13757-x.

## Background

China is one of the countries with the highest rate of aging, and the situation of aging in China is severe [1]. As of 2019, the population of China accounted for 18% of the global population. Among them, the number of people aged 65 or older reached 165 million, and the number of people aged 80 or older reached 26 million. By 2050, it is expected that the total number of people over 65 years of age in China will reach 365 million [2]. The intensified aging of the population makes the existing elderly care and medical service resources unable to meet the growing needs of the elderly [3]. Responding actively to population ageing should be a long-term strategy of the country [4], for which healthy ageing was proposed and gradually became the theme of the times [2]. In 2015, the WHO defined healthy ageing in its *World report on aging and health* as “the process of developing and maintaining the functional ability that enables well-being in older age” [5]. Healthy ageing is a government goal and an important condition for the national health level and national economic and social development. The Chinese government has formulated the “Health China 2030” planning outline, which places health as a strategic priority for development and is an important manifestation of the government’s active response to population aging and achievement of healthy ageing [6].

A comprehensive understanding of the healthy ageing trajectory of individuals and its factors is of great significance for health development strategies. One of the most important issues is the disparity in healthy ageing of individuals living in rural and urban areas. Research on the current status and trajectory of healthy ageing between urban-rural areas in China is lacking but important. The rural/urban residence indicates household living region and is defined by National Bureau of Statistics of the People’s Republic of China. Regarding the socioeconomic background, compared to other countries, China has a large urban-rural disparity in terms of economic income [7], with that the income of the urban population is 2.5 times that of the rural population in 2021, and the per capita disposable income of urban residents and rural residents is RMB 47,412 and RMB 18,931 respectively [8]; a larger proportion of older people and a faster aging process in rural areas than in urban areas, with the proportion of people aged 60 and over reaching 20.04% and the proportion of people aged 65 and over reaching 13.82% in rural areas according to China Rural Revitalization Survey (CRRS); and a lower level of education in rural areas than in urban areas due to geographical location and hukou policies, which are special identifiers in China and affect many aspects of life such as buying a house, buying a car, children’s school enrollment and other welfare [9]. In terms of family structure, a large number of left-behind children (whose parents went to cities to earn money) and older empty nesters have been increasingly common in rural areas due to population mobility brought about by socioeconomic transformation [10, 11]; there are differences between urban and rural populations in terms of the frequency of contact with children [12] and economic interactions with children [13]. In terms of personal life, rural populations differ from urban populations in smoking rates [14], alcohol consumption rates [15], and utilization of medical checkups [16]. In addition, there are differences in healthcare resources between urban and rural areas [17]. Numerous studies have demonstrated differences between urban and rural areas in China, but it is unclear what the current status and trajectory of healthy ageing are in urban and rural areas, respectively, and whether disparity of healthy ageing exists between them.

Middle-aged adults are the group of people aged 45 to 65 years who are in a special period of transition to old age. Compared to most of previous studies on healthy ageing [2, 18, 19], we included a sample of middle-aged adults to analyze the trajectory of healthy ageing. Some studies have analyzed healthy ageing among middle-aged adults, but have not focused on urban-rural disparity [20].

To better study population aging, many studies have focused on defining and measuring healthy ageing and established a healthy ageing score [21, 22], which provides a good reference for our study. The aim of this study is to assess whether there are disparities in healthy ageing trends between urban and rural areas. We further explore factors associated with healthy ageing in rural and urban areas, respectively, to provide targeted intervention suggestions.

## Methods

### Data source and sample selection

Data used for this study were from the China Health and Retirement Longitudinal Study (CHARLS), which was conducted by the National School of Development of Peking University. The survey was conducted for 4 waves (2011, 2013, 2015 and 2018). With a multistage stratified probability-scale proportional sampling method to select interviewees for Chinese residents aged 45 and older, and one-on-one interviews with a structured questionnaire to collect high-quality data, CHARLS has been widely used to explore issues related to healthy ageing. The response rate for the first wave (2011) of CHARLS was 80.5%, and the total sample size in 2011 was 17,708, who were followed up every 2 years with repeat surveys. The data included individual weighting variables to ensure a nationally representative survey sample [23]. Individual questionnaires included basic demographic and household transfer information; health status and functioning; health care and insurance; employment, retirement, and pensions; income and consumption; and household assets. Details of the sampling method and questionnaire can be found on the official website (http://charls.pku.edu.cn/). The Biomedical Ethics Review Board of Peking University approved CHARLS, and all participants were required to provide written informed consent. The ethical approval number is IRB00001052–11015. A more detailed description of the objectives and methods of CHARLS has been reported elsewhere [24].

Four waves of survey data were used for this study. Respondents aged less than 45 years old in 2011 and individuals lost to follow-up survey were excluded. In addition, given the accuracy of the healthy ageing index, individuals with a denominator of less than 26 (80% out of the total 42 items) were excluded from the study [25]. A total of 9402 respondents who completed all four data points were enrolled in the final analysis, with 6167 respondents (65.6%) living in rural areas, and 3235 respondents (34.4%) living in urban areas. According to the data of the Sixth National Census (2010) in China, the urban-rural ratio of population aged ≥45 years old is 1:1.48. Therefore, respondents included in our analysis basically conform to the urban-rural distribution of the national population in China.

### Variables

The healthy ageing score was the outcome measure in this study. Based on the WHO framework and previous studies [26–28], we reviewed the information included in the CHARLS survey and identified 32 items (Supplementary Table S1) that might indicate the underlying concept of healthy ageing. The selected items mainly focused on physical and cognitive function, pain, hearing/eyesight problems and memory problem, which strongly influence daily health performance. The selected items were dichotomized into binary variables (0 = presence of difficulties, 1 = absence of difficulties). Item response theory (IRT) modeling was used to incorporate 32 items and estimate latent trait scores for respondents on the basis of the unidimensionality assumption. IRT models can account for variation in response patterns, difficulty and differentiation of the items, and generate corresponding latent trait scores to reflect such variation. To improve the interpretation of the results, the latent trait scores were transferred into a range between 0 and 100:

- $$\mathrm{HAI}=\frac{\mathrm{x}-\min }{\max -\min}\times 100$$
- HAI indicates the final healthy ageing score; X is the latent trait score calculated by IRT. Min and max represent the minimum and maxmum latent trait score generated by IRT, respectively. We also calculated the empirical reliability and marginal reliability with 0.802 and 0.737, respectively, which presents well-performed reliability of the sum scores [29].

The primary independent variable is the residence of respondents (0 = rural, 1 = urban), indicating the living region of the household, and is defined by the National Bureau of Statistics of the People’s Republic of China.

The covariates in this study include respondents’ socioeconomic background (age, gender, marital status, educational level, household per capita consumption, public health insurance coverage, current work status and chronic conditions), family characteristics (whether gives care to grandchildren, whether lives near children, weekly contact with children, gave money to children, received money from children) and lifestyle (alcohol intake, smoking status, social participation and physical examination). The definition and classifications are detailed in Supplementary Table S2.

### Statistical analysis

In description analysis of the respondents’ baseline characteristics, “number (percentage)” was used for the description of binary or categorical variables, and “mean ± standard deviation (SD) “ and “median (percentages)” were used for the description of continuous variables with normal distribution and abnormal distribution, respectively. The significance of the variances of binary or categorical variables were analyzed using χ2 or Fisher’s exact test and those of continuous variables were analyzed using t-tests or a non-parametric equivalent (Wilcoxon rank test). Bonferroni corrections were made for multiple comparisons (Supplementary Table S3).

We adopted the general additive models (GAM) to fit the regression for healthy ageing score on survey wave in rural and urban areas. GAM extends the generalized linear model, in which the predictor function may contain one or more user-specified sums of smooth functions of the covariates plus a conventional parametric component of the linear predictor. With the cubic spline smoothing function to control for the confounding factors, an additional smoothing function of survey wave was constructed to filter out the trends of outcomes, and could reveal the trend variance between different groups [30–32].

A latent growth mixture model (LGMM) was applied to classify the trajectory of the healthy ageing score of the respondents across 4 survey waves and to test predictors of membership in these classes [33, 34]. The LGMM is efficient at modeling the variation in growth parameters that incorporate information from multiple indicators (repeated measures of an outcome). Furthermore, LGMM analysis does not assume a single population and can test for the presence of multiple groups or classes of individuals who represent distinct multivariate normal distributions [35–37]. We compared one- to four-class unconditional LGMMs and assessed the relative fit with conventional indices. To determine the appropriate class solution, we examined the Bayesian information criterion (BIC), the Akaike information criterion (AIC), entropy values, and the Lo-Mendell-Rubin likelihood ratio test (LRT).

After trajectory groups of healthy ageing were identified, a multinomial logistic regression model (MLRM) was further performed to investigate the effect of rural/urban areas on trajectory type in middle-aged and older adults, with covariates in 2011 controlled. We further explored factors associated with trajectory type in rural and urban areas, respectively, through MLRM. In addition, we repeat MLRM among respondents aged ≥65 years old and < 65 years old separately as sensitivity analysis. The relative risk ratio (RRR) and confidence interval (CI) were calculated, with an RRR < 1 indicating a higher likelihood of being healthy. Considering the covariates may be time-variant, we additionally applied random-effects model, to assess the impact of rural/urban residence on healthy ageing score.

The *P* values were two-sided, and an alpha level of 0.05 was used to define statistical significance. The data were analyzed using Stata (version 15) and R version 3.6.3 (R Foundation for Statistical Computing, Vienna, Austria).

## Results

Table 1 shows the descriptive statistics of the variables used in this study for both the rural and urban samples. Of all respondents, rural older adults accounted for 65.59% (6167). Urban-rural respondents significantly differed in socioeconomic background, family characteristics, and personal lifestyle. In terms of socioeconomic background, more than four-fifths of rural older adults were still working, a significantly higher percentage than that among urban older adults (three-fifths). In addition, the vast majority (93.32%) of rural older adults did not have upper secondary education, with a significant higher proportion than that among urban older adults (81.02%). Through Bonferroni correction, rural respondents had a significant higher proportion of low and low-to-middle consumption than urban respondents, and urban respondents had a significant higher proportion of high and middle consumption than rural respondents. In terms of family characteristics, a significantly higher proportion of urban older adults cared for grandchildren (57.26%), co-resided with children (93.72%), and were in contact with children (95.71%) than rural older adults (48.34, 91.47, and 91.02% respectively). However, only 27.55% of urban respondents received financial support from children, which was much lower than the proportion of rural respondents (39.10%). In terms of personal lifestyles, rural respondents had a less healthy lifestyle than urban respondents in terms of smoking, drinking, and social interactions. The proportion of currently smoking respondents was significantly higher in rural areas than that in urban. In addition, more than half (55.52%) of urban older adults had physical examinations within the past 2 years, which was higher than that of rural respondents (47.25%).

**Table 1:** Baseline descriptions (*N* = 9402)

	Rural(*n* = 6167)	Urban(*n* = 3235)	*P* value
**1.socioeconomic background**			
**Age**	58.00 (45.00–93.00)	57.00 (45.00–95.00)	0.239
**Gender**			0.066
Male	2818(45.69%)	1414(43.71%)	
Female	3349(54.31%)	1821(56.29%)	
**Educational level**			< 0.001
Less than lower secondary	5755(93.32%)	2621(81.02%)	
upper secondary & vocational	396(6.42%)	509(15.73%)	
tertiary	16(0.26%)	105(3.25%)	
**Marital status**			0.653
Divorced or widowed	661(10.72%)	337(10.42%)	
Married	5506(89.28%)	2898(89.58%)	
**Household per capita consumption**			
Low	2203 (40.99%)	590 (21.91%)	< 0.001
Low-to-middle	1581 (29.42%)	617 (22.91%)	
Middle	1053 (19.59%)	770 (28.59%)	
High	537 (9.99%)	716 (26.59%)	
**Public health insurance coverage**			< 0.001
Not covered	295(4.80%)	278(8.62%)	
Covered	5857(95.20%)	2946(91.38%)	
**Current work status**			< 0.001
Not working	1110(18.06%)	1382(42.92%)	
Working	5036(81.94%)	1838(57.08%)	
**Chronic condition**			0.317
None	1810(29.35%)	908(28.07%)	
Yes	1844(29.90%)	962(29.74%)	
Morbidity	2513(40.75%)	1365(42.19%)	
**2.Family characteristics**			
**Gave care to grandchildren**			< 0.001
None	2289(51.66%)	854(42.74%)	
Yes	2142(48.34%)	1144(57.26%)	
**Live near children**			< 0.001
None	516(8.53%)	199(6.28%)	
Yes	5530(91.47%)	2969(93.72%)	
**Weekly contact with children**			< 0.001
None	544 (8.98%)	136 (4.29%)	
Yes	5515(91.02%)	3035 (95.71%)	
**Gave money to children**			0.029
None	4980(81.04%)	2673(82.88%)	
Yes	1165(18.96%)	552(17.12%)	
**Received money from children**			< 0.001
None	3745(60.90%)	2341(72.45%)	
Yes	2404(39.10%)	890(27.55%)	
**3.Lifestyle**			
**Alcohol intake**			0.524
Do not drink	4161(67.48%)	2204(68.13%)	
Drink	2005(32.52%)	1031(31.87%)	
**Smoking status**			< 0.001
Never	3750(62.02%)	2094(65.60%)	
Quit now	455(7.53%)	254(7.96%)	
Still	1841(30.45%)	844(26.44%)	
**Social participation**			< 0.001
None	3325(53.92%)	1566(48.42%)	
Yes	2841(46.08%)	1668(51.58%)	
**Physical Examination**			< 0.001
None	3252(52.75%)	1438(44.48%)	
Yes	2913(47.25%)	1795(55.52%)	

*Note*: Age was described by median (min-max), and the variance was tested by Wilcoxon rank test because of the abnormal distribution

**Fig. 1:**
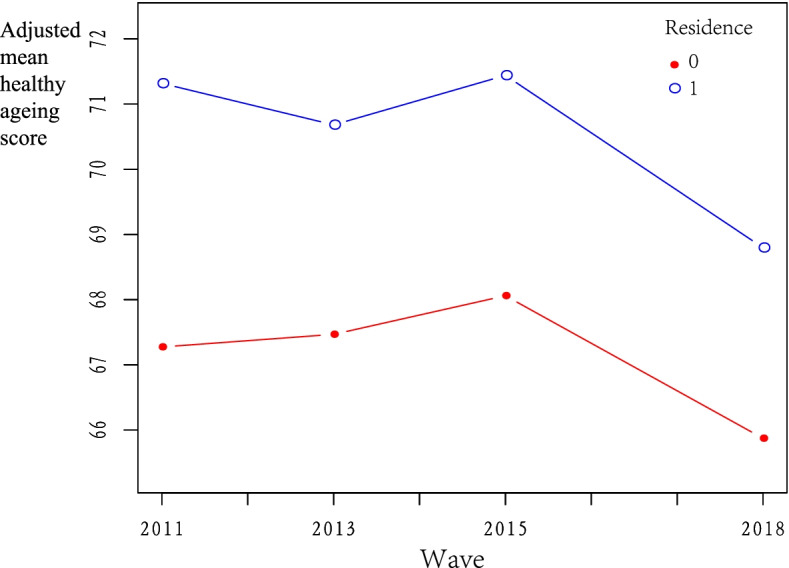
Smooth curve fitting for healthy ageing score across 4 waves based on generalized additive model. 0 = rural; 1 = urban

Table 2 shows that the average healthy ageing score gradually decreased from 68.17 in 2011 to 60.38 in 2018 among the total respondents, from 70.83 in 2011 to 62.88 in 2018 among urban respondents, and from 66.77 in 2011 to 59.07 in 2018 among rural respondents. The average healthy ageing score of rural respondents was lower than that of urban respondents in each wave. After controlling all the covariates, smooth curving based on GAM presented that the adjusted mean healthy ageing score in rural areas was significantly lower than that in urban areas, and significant declines of healthy ageing score during 2015–2018 were observed in both rural and urban areas (see Fig. 1).

**Table 2:** Description of healthy aging scores within different groups (Mean ± SD)

	2011	2013	2015	2018
Overall	68.17 ± 15.69	66.66 ± 15.39	64.63 ± 16.06	60.38 ± 15.63
Rural	66.77 ± 15.65	65.46 ± 15.29	63.20 ± 16.03	59.07 ± 15.55
Urban	70.83 ± 15.42	68.96 ± 15.30	67.34 ± 15.74	62.88 ± 15.47

**Fig. 2:**
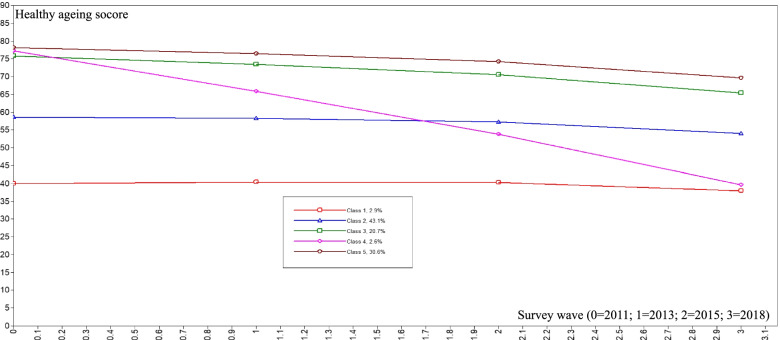
Trajectory of healthy ageing score by latent growth mixture model. The definition of the classes: Class 1, continuing-low; Class 2, continuing-middle; Class 3, continuing-middle-to-high; Class 4, significantly-declining; Class 5, continuing-high

The results of the LGMM are shown in Table 3. We compared two- to six-class unconditional models for healthy ageing scores after adjusting for age and gender, and examined the BIC, AIC entropy values and LRT. We sought a model with lower values for the criterion indices, higher entropy values, and LRT *P* value. The results suggested that a five-class solution was the best. Then, we estimated the means of each class in every survey wave and defined the five classes as “continuing-high”, “continuing-middle-to-high”, “continuing-middle”, “continuing-low”, and “significantly-declining” to represent the trajectories of healthy ageing. As shown in Fig. 2, there was an obviously downward trend in the overall health status with age. The results of the distribution of healthy ageing trajectories within different groups (Table 4) show that rural respondents had a higher proportion of significantly-declining trajectory type than urban respondents (1.69% vs 1.02%) and a lower proportion of continuing-high and continuing-middle-to-high trajectory types (30.97% vs 34.37, 17.17% vs 24.88%).

**Table 3:** Fit indices for two- to four-class growth mixture models for healthy aging score

	AIC	BIC	Entropy	Lo-Mendell-Rubin test *P* value
**2 classes**	293,462.738	293,584.265	0.428	< 0.001
**3 classes**	293,304.499	293,454.622	0.590	< 0.001
**4 classes**	293,183.644	293,362.361	0.635	< 0.001
**5 classes**	293,130.617	293,337.929	0.720	< 0.001
**6 classes**	293,093.300	293,329.206	0.568	0.240

*Note*: Healthy ageing scores was adjusted for age and gender

*AIC* Akaike information criterion, *BIC* Bayesian information criterion

**Table 4:** Distribution of healthy aging trajectory within different groups, N (%)

	Overall	Rural	Urban
**Continuing-high**	3022 (32.14%)	1910 (30.97%)	1112 (34.37%)
**Continuing-middle-to-high**	1864 (19.83%)	1059 (17.17%)	805 (24.88%)
**Continuing-middle**	4224 (44.93%)	2978 (48.29%)	1246 (38.52%)
**Continuing-low**	155 (1.65%)	116 (1.88%)	39 (1.21%)
**Significantly-declining**	137 (1.46%)	104 (1.69%)	33 (1.02%)

MLRM was conducted to investigate the effect of rural/urban residence on trajectory type of healthy ageing score in older adults with potential confounders adjusted. Urban residence was significantly associated with a higher likelihood of being healthy (for [continuing-low/continuing-high]: β = − 1.17, RRR = 0.31, *P* < 0.001, 95% CI = 0.18–0.53; and for [continuing-middle/continuing-high]: β = − 0.53, RRR = 0.59, *P* < 0.001, 95% CI = 0.49–0.71). The details are shown in Table 5.

**Table 5:** Multinomial logistic regression of trajectory type (Reference: continuing-high group)

	Continuing-low		Continuing-middle		Continuing-middle-to-high		Significantly-declining	
	RRR(95%CI)	*P* value	RRR(95%CI)	*P* value	RRR(95%CI)	*P* value	RRR(95%CI)	*P* value
**Residence**								
Urban	0.31(0.18,0.53)	< 0.001	0.59(0.49,0.71)	< 0.001	1.09(0.88,1.36)	0.432	0.66(0.39,1.13)	0.133
**Educational level**								
Upper secondary & vocational	0.42(0.17,1.02)	0.054	0.28(0.21,0.38)	< 0.001	0.39(0.28,0.56)	< 0.001	0.58(0.27,1.26)	0.168
Tertiary	–	–	0.12(0.05,0.29)	< 0.001	0.21(0.08,0.56)	0.002	–	–
**Marital status**								
Married	1.49(0.67,3.30)	0.324	0.74(0.55,1.00)	0.049	0.69(0.48,0.98)	0.041	0.62(0.29,1.31)	0.207
**Household per capita consumption**								
Low-middle	1.50(0.87,2.58)	0.142	1.03(0.85,1.25)	0.750	1.20(0.94,1.52)	0.148	1.60(0.91,2.82)	0.106
Middle-high	1.64(0.92,2.91)	0.093	0.95(0.77,1.17)	0.664	1.10(0.84,1.43)	0.481	1.24(0.65,2.36)	0.508
High	1.19(0.57,2.51)	0.643	1.00(0.77,1.29)	0.977	1.46(1.07,2.00)	0.018	1.47(0.69,3.12)	0.313
**Public health insurance coverage**								
Covered	2.06(0.70,0.08)	0.189	1.05(0.75,1.47)	0.771	1.12(0.75,1.69)	0.571	0.62(0.29,1.37)	0.241
**Current work status**								
Working	0.11(0.07,0.17)	< 0.001	0.54(0.44,0.67)	< 0.001	0.59(0.46,0.75)	< 0.001	0.49(0.28,0.85)	0.010
**Chronic condition**								
Yes	2.98(1.24,7.17)	0.015	1.71(1.40,2.10)	< 0.001	0.73(0.57,0.92)	0.007	1.05(0.59,1.89)	0.865
Morbidity	10.80(4.84,24.10)	< 0.001	4.08(3.35,4.97)	< 0.001	0.70(0.55,0.89)	0.003	1.67(0.96,2.90)	0.069
**Gave care to grandchildren**								
Yes	0.63(0.41,1.00)	0.045	1.11(0.95,1.30)	0.169	1.04(0.86,1.27)	0.663	1.00(0.63,1.58)	1.000
**live near children**								
Yes	1.38(0.54,3.49)	0.499	0.905(0.67,1.22)	0.513	1.26(0.84,1.90)	0.259	0.96(0.39,2.41)	0.936
**Weekly contact with children**								
Yes	0.72(0.33,1.55)	0.397	1.00(0.74,1.34)	0.984	0.82(0.56,1.21)	0.322	0.87(0.37,2.07)	0.760
**Gave money to children**								
Yes	0.52(0.28,0.96)	0.037	0.79(0.65,0.95)	0.011	0.96(0.76,1.20)	0.699	0.74(0.41,1.33)	0.308
**Received money from children**								
Yes	1.05(0.68,1.63)	0.823	1.11(0.94,1.30)	0.218	1.06(0.87,1.30)	0.554	0.64(0.39,1.07)	0.087
**Alcohol intake**								
Drink	0.44(0.26,0.75)	0.003	0.40(0.34,0.47)	< 0.001	0.21(0.16,0.26)	< 0.001	0.52(0.32,0.87)	0.012
**Smoking status**								
Quit now	0.38(0.20,0.72)	0.003	0.14(0.11,0.18)	< 0.001	0.03(0.02,0.06)	< 0.001	0.31(0.15,0.68)	0.004
Still	0.25(0.15,0.41)	< 0.001	0.12(0.10,0.15)	< 0.001	0.03(0.02,0.05)	< 0.001	0.28(0.17,0.47)	< 0.001
**Social participation**								
Yes	0.29(0.18,0.47)	< 0.001	0.88(0.75,1.02)	0.090	1.12(0.93,1.36)	0.237	1.14(0.72,1.79)	0.575
**Physical examination**								
Yes	0.67(0.43,1.03)	0.065	0.85(0.72,0.99)	0.035	0.81(0.66,0.98)	0.030	0.73(0.46,1.15)	0.170

*RRR* Relative Risk Ratio, *CI* Confidence intervals

Table 6 shows that education, marriage, work status, chronic disease status, alcohol intake, smoking status, and social participation are common influences on healthy ageing trajectories in both urban and rural areas. In addition, income, and physical examination were associated with healthy ageing trajectory in rural areas; while gave care to grandchildren, gave money to children were related to healthy ageing trajectory in urban areas.

**Table 6:** Multinomial logistic regression of trajectory type in rural and urban areas (Reference: continuing-high group)

	Rural (n = 6167)				Urban (n = 3235)			
	Continuing-low	Continuing-middle	Continuing-middle-to-high	Significantly-declining	Continuing-low	Continuing-middle	Continuing-middle-to-high	Significantly-declining
**Educational level**								
Upper secondary & vocational	0.23(0.05,1.01)	0.29(0.20,0.42)***	0.29(0.17,0.50)***	0.70(0.27,1.83)	1.24(0.35,4.33)	0.27(0.17,0.43)***	0.48(0.30,0.79)**	0.43(0.12,1.56)
Tertiary	–	0.20(0.02,2.12)	–	–	–	0.12(0.05,0.33)***	0.20(0.07,0.57)**	–
**Marital status**								
Married	3.09(1.05,9.10)*	0.92(0.02,2.12)	1.01(0.70,1.72)	0.70(0.30,1.67)	0.35(0.10,1.28)	0.44(0.24,0.82)*	0.29(0.15,0.56)***	0.48(0.10,2.31)
**Household per capita consumption**								
Low-middle	1.76(0.96,3.22)	1.09(0.87,1.35)	1.22(0.92,1.63)	1.84(0.97,3.49)	0.96(0.27,3.47)	0.82(0.54,1.24)	1.02(0.63,1.63)	0.87(0.25,3.01)
Middle-high	2.25(1.16,4.34)*	1.01(0.78,1.30)	1.19(0.85,1.67)	1.53(0.71,3.26)	0.79(0.23,2.68)	0.87(0.59,1.28)	0.92(0.58,1.46)	0.83(0.26,2.72)
High	1.28(0.52,3.15)	0.97(0.69,1.35)	1.48(0.97,2.26)	1.21(0.43,3.40)	0.77(0.18,3.27)	1.04(0.67,1.61)	1.37(0.83,2.26)	1.63(0.49,5.39)
**Public health insurance coverage**								
Covered	1.83(0.51,6.62)	0.92(0.59,1.43)	1.19(0.67,2.13)	0.97(0.29,3.32)	3.24(0.39,27.06)	1.39(0.82,2.35)	1.16(0.65,2.07)	0.42(0.14,1.24)
**Current work status**								
Working	0.07(0.04,0.12)***	0.44(0.33,0.59)***	0.59(0.41,0.84)**	0.33(0.17,0.65)**	0.32(0.12,0.85)*	0.67(0.49,0.91)*	0.56(0.40,0.80)**	0.78(0.32,1.90)
**Chronic condition**								
Yes	3.47(1.26,9.59)*	1.67(1.32,2.12)***	0.67(0.51,0.90)**	1.09(0.54,2.20)	1.18(0.16,8.83)	1.82(1.23,2.69)**	0.89(0.58,1.35)	1.05(0.36,3.05)
Morbidity	10.72(4.15,27.74)***	4.08(3.24,5.14)***	0.62(0.46,0.83)**	1.88(0.97,3.63)	9.91(2.17,45.17)**	4.01(2.76,5.83)***	0.83(0.55,1.26)	1.33(0.48,3.69)
**Gave care to grandchildren**								
Yes	0.84(0.51,1.39)	0.84(0.52,1.39)	0.91(0.72,1.16)	1.07(0.62,1.85)	0.24(0.08,0.70)**	1.20(0.89,1.60)	1.34(0.96,1.88)	0.91(0.39,2.13)
**live near children**								
Yes	1.01(0.38,2.69)	1.01(0.38,2.69)	1.19(0.74,1.94)	1.29(0.42,3.98)	929,277.5(0,-)	1.28(0.67,2.43)	1.55(0.72,3.33)	0.51(0.11,2.48)
**Weekly contact with children**								
Yes	0.77(0.32,1.85)	0.77(0.32,1.85)	0.82(0.53,1.27)	0.61(0.25,1.50)	0.57(0.10,3.13)	1.09(0.55,2.18)	0.88(0.40,1.95)	–
**Gave money to children**								
Yes	0.62(0.31,1.85)	0.62(0.31,1.24)	1.08(0.81,1.44)	0.89(0.45,1.73)	0.31(0.07,1.43)	0.68(0.48,0.96)*	0.75(0.51,1.12)	0.45(0.13,1.57)
**Received money from children**								
Yes	0.90(0.54,1.47)	0.90(0.54,1.47)	1.04(081,1.33)	0.68(0.38,1.22)	1.70(0.66,4.36)	1.21(0.89,1.65)	1.13(0.79,1.62)	0.49(0.16,1.51)
**Alcohol intake**								
Drink	0.32(0.17,0.60)***	0.32(0.17,0.60)***	0.18(0.13,0.25)***	0.53(0.29,0.96)*	0.96(0.35,2.64)	0.40(0.30,0.55)***	0.24(0.16,0.36)***	0.52(0.19,1.40)
**Smoking status**								
Quit now	0.47(0.22,0.98)*	0.47(0.22,0.98)***	0.04(0.02,0.08)***	0.42(0.17,1.00)*	0.25(0.06,0.99)*	0.09(0.05,0.14)***	0.03(0.01,0.07)***	0.10(0.01,0.80)*
Still	0.26(0.14,0.47)	0.26(0.14,0.47)***	0.03(0.02,0.04)***	0.27(0.15,0.51)***	0.26(0.09,0.78)*	0.13(0.09,0.18)***	0.04(0.02,0.06)***	0.28(0.11,0.73)**
**Social participation**								
Yes	0.32(0.19,0.56)***	0.32(0.18,0.56)	1.02(0.81,1.30)	1.13(0.66,1.93)	0.17(0.05,0.53)**	0.71(0.53,0.95)*	1.23(0.88,1.73)	1.10(0.47,2.57)
**Physical examination**								
Yes	0.65(0.40,1.07)	0.65(0.40,1.07)*	0.84(0.66,1.06)	0.73(0.42,1.25)	0.62(0.24,1.59)	0.92(0.68,1.24)	0.75,0.53,1.05)	–

Note: *P < 0.05; **P < 0.01; ***P < 0.001. “--” indicates abnormal values caused by small sample size

Table 7 shows that the results of MLRM for respondents aged ≥65 years old and < 65 years old were consistent with the main analysis (Table 5), which validated our conclusions.

**Table 7:** Subgroup Analysis by Age

	Continuing-low		Continuing-middle		Continuing-middle-to-high		Significantly-declining	
	RRR(95%CI)	*P* value	RRR(95%CI)	*P* value	RRR(95%CI)	*P* value	RRR(95%CI)	*P* value
**≥65 years old**								
Urban	0.20(0.05,0.87)	0.032	0.45(0.30,0.69)	< 0.001	1.23(0.74,2.06)	0.424	0.46(0.13,1.57)	0.214
**< 65 years old**								
Urban	0.33(0.18,0.59)	< 0.001	0.62(0.50,0.76)	< 0.001	1.07(0.83,1.37)	0.603	0.71(0.39,1.31)	0.273

*Note*: Adjusting factors include educational level, marital status, household per capita consumption, public health insurance coverage, current work status, chronic condition, gave care to grandchildren, live near children, weekly contact with children, gave money to children, received money from children, alcohol intake, smoking status, social participation, physical examination

RRR: Relative Risk Ratio; CI: Confidence intervals

We additionally apply random-effects model to assess the impact of rural/urban residence on healthy ageing score accounting for time-varying covariates. The results (Table 8) also shows that urban respondents were more likely to have higher healthy ageing score than rural respondents (β = 4.19; *P* < 0.001).

**Table 8:** The impact of rural/urban areas on healthy ageing score based on random-effects model

	β (95%CI)
**Residence**	
Urban	4.19(3.62,4.76)***
**Age**	−0.40(−0.43,-0.37)***
**Gender**	
Female	6.42(−7.17,-5.67)***
**Marital status**	
Married	0.90(0.23,1.56)**
**Urban or rural**	
Urban	4.19(3.62,4.76)***
**Educational level**	
Upper secondary & vocational	4.45(3.56,5.35)***
Tertiary	8.59(6.63,10.54)***
**Household per capita consumption**	
Low-middle	0.30(−0.19,0.79)
Middle-high	−0.00(− 0.50,0.49)
High	− 0.14(− 0.64,0.36)
**Public health insurance coverage**	
Covered	−0.17(− 0.95,0.61)
**Current work status**	
Working	3.38(2.89,3.88)***
**Chronic condition**	
Yes	−4.10(−4.71,-3.49)***
Morbidity	−9.41(−10.01,-8.80)***
**Gave care to grandchildren**	
Yes	0.14(−0.23,0.51)
**Live near children**	
Yes	1.10(0.68,1.52)***
**Weekly contact with children**	
Yes	1.17(0.62,1.71)***
**Gave money to children**	
Yes	0.05(−0.31,0.40)
**Received money from children**	
Yes	−0.16(− 0.54,0.23)
**Smoking status**	
Quit now	−1.78(−2.58,-0.99)***
Still	−0.38(−1.12,0.36)
**Alcohol intake**	
Drink	0.30(−0.19,0.79)
**Social participation**	
Yes	1.54(1.18,1.90)***
**Physical examination**	
Yes	0.41(0.06,0.76)*

Note: *P < 0.05; **P < 0.01; ***P < 0.001

## Discussion

To our best knowledge, this is the first study to assess the disparity of healthy ageing trajectory between rural and urban areas in China. We measured the level of healthy ageing by an index (healthy ageing score) that integrates the physiological, psychological, and cognitive functional states of the middle-aged and older population through IRT. Through a latent growth mixture model, a decreasing trend of healthy ageing score was found, however, the decreasing magnitudes differed. We further found the distribution in different trajectory groups was significantly varied between urban and rural areas, with a higher proportion of significantly-declining and a lower proportion of continuing-high and continuing-middle-to-high trajectory types in rural areas than those in urban areas. Multinomial logistics regression model further indicated that rural populations were more likely to develop lower level of healthy ageing.

A study analyzed healthy ageing scores in eight countries with mean ages ranging from 61.5 to 77.5 and healthy ageing scores ranging from 56.8 to 76.9, with a combined country score of 67.5 and an overall mean age of 62.9 [28]. Our study showed that the baseline population (mean age 58.2, median 58) had a mean healthy ageing score of 68.2, which is within a reasonable range. This study indicated that the trajectory of declining health status is irreversible for older adults along with aging. However, we found several healthy ageing trajectories with different levels of health status and different slopes, which indicated that the loss of health can be controlled, or at least delayed.

Healthy ageing is a prominent objective in the development of a country, and rural-urban disparities are an essential obstacle to overcome. In China, the disparity of healthy ageing between rural and urban areas is significant. Although the trajectory of declining health status is irreversible for older adults, the risk of unsuccessful aging is much higher in rural areas than in urban areas, which could be indicated by the higher risk of being enrolled in the “continuing-low” and “continuing-middle” healthy ageing trajectory group in rural areas.

We further identified factors associated with healthy ageing trajectory in rural and urban areas respectively and mainly concentrated on the “continuing-low” and “continuing-middle” groups. We found that higher levels of education are more likely to be healthy than those who have not received higher education. Relevant studies have shown that higher levels of education are associated with longer lifespans and delayed disease onset [38]. People with higher education may have higher socioeconomic status, resulting in increased life satisfaction [39]. People who are still working are more likely to be healthy. Although the work of the rural population is mainly engaged in agricultural production, and this work is continuous, unlike urban workers who have a time point for retirement age, we found its consistent impacts on healthy ageing trajectory in both rural and urban areas, which may be related to the fact that those who work are responsible for the family and are generally a source of income, and there is a significant positive gradient between life satisfaction and finances [40]. Multi-morbidity is associated with low healthy ageing level in both rural and urban areas, which is more and more common in China, and brings great challenges to the medical system and health managers. Therefore, strengthening prevention and standardized management of chronic diseases is in urgent need [1, 41]. Alcohol intake and smoking status are associated with likelihood of being healthy, which is not consistent with many previous studies. This may be caused by that the grouping methods of these two variables are rough due to the data limitation, and actual frequencies or intensities of drinking and smoking have not been measured in this study. Further studies should be conducted to assess whether and how drinking and smoking affect healthy ageing. People who are involved in social life are more likely to be healthy, related to the environmental factors in which they live [42–44]. We should encourage older people to participate more in social activities to develop a good environment and outlook. In addition, based on previous studies, we found that there are differences between urban and rural areas in terms of basic health status [45] and resources that can be accessed for medical services [46], which are the reasons for the differences in healthy ageing between urban and rural areas.

By clarifying the differences in healthy ageing between urban and rural areas and finding the factors that influence the differences between urban and rural areas, we can take more effective measures to promote healthy ageing. To alleviate the disparity in healthy ageing between urban and rural areas, China has established a relatively well-developed social security system [47]. However, this system still separates urban and rural areas and maintains a “dual-track” operation, and inequities remain. For this reason, it is important to balance the urban and rural economies, promote rural revitalization, and establish a national social security network to maintain efficiency and equity and alleviate the urban-rural healthy ageing gap [48, 49]. Rural areas have low population density and large distances between homes and services [50]. Health care facilities are difficult to operate, and the level of population health utilization is low. For this reason, rural infrastructure should be well developed. Finally, the older population should be encouraged to participate more in social activities, and a good social atmosphere should be formed by setting up clubs, promoting a diversity of activities, strengthening publicity and education for the older population in urban and rural communities.

There are some limitations of this study. First, because our study used CHARLS data from respondents’ self-administered questionnaires, the results of the questionnaire depend on the respondents, and respondents may inevitably experience recall bias when filling out the questionnaire due to subjectivity (such as “self-reported pain” to calculate healthy ageing score), unclear recall (such as “household per capita consumption”) and other reasons. Second, as a retrospective study, our study proved that the disparity of healthy ageing trajectory exists between rural and urban areas in China, and found some interventions to promote healthy ageing in rural and urban areas, respectively. Nevertheless, we could not prove more evidence about the systematic reasons and mechanisms causing this disparity. Third, due to the limitation of data, some variables could not be classified into more detailed groups, therefore, we may not be able to provide more accurate suggestions. More studies associated with how to promote healthy ageing and reduce disparity of healthy ageing trajectory between rural and urban areas in China should be further conducted in the future. Fourth, only respondents completed surveys at all time points were included in our analysis, which leads to sample loss. However, through multiple analyzing models and sensitivity analysis, our conclusion associated with rural-urban disparity in healthy ageing is relatively robust. Finally, the current understanding of whether healthy ageing score, as an outcome measure, is sensitive to change is limited. Moreover, it is difficult to interpret the change. In the future, research should be conducted to establish the psychometric properties of the healthy ageing score.

## Conclusion

Our study used data on middle-aged and older adults from 2011 to 2018 to examine the impact of urban-rural residence on the healthy ageing trajectory. We found that the healthy ageing level showed an obviously downward trend, and this downward trajectory differed between urban and rural areas, with the rural populations were more likely to develop low level of healthy ageing. Prevention and standardized management of chronic diseases should be enhanced, and social participation should be encouraged to promote healthy ageing. The policy inclination and resource investment should be enhanced to reduce disparity in healthy ageing between urban and rural areas in China.

## Supplementary Information

**Additional file 1: Table S1.** Items to calculate healthy ageing score. **Table S2.** Variable description. **Table S3.** Bonferroni corrections of baseline descriptions.

## Data Availability

All the original data could be obtained from the official website of CHARLS (http://charls.pku.edu.cn/) and Harmonized CHARLS (www.g2aging.org). The deidentified analysis dataset is available to other researchers and others upon request by emailing the corresponding author.

## Abbreviations

CHARLS: China Health and Retirement Longitudinal Study
IRT: Item response theory
MLRM: Multinomial logistics regression model
BIC: Bayesian information criterion
AIC: Akaike information criterion
LRT: Lo-Mendell-Rubin likelihood ratio test
LGMM: Latent growth mixture model
RRR: Relative Risk Ratio
CI: Confidence intervals
SD: Standard deviation
