# Supplementary material for: Urban-rural disparities in the healthy ageing trajectory in China: a population-based study
Source: BMC Public Health. 2022 Jul 23;22:1406. doi: 10.1186/s12889-022-13757-x (PMC9308310; doi:10.1186/s12889-022-13757-x)
Supplement: Supplementary file 1 — Additional file 1: Table S1. Items to calculate healthy ageing score. Table S2. Variable description. Table S3. Bonferroni corrections of baseline descriptions. [file 12889_2022_13757_MOESM1_ESM.docx]

**Table S1.** Items to calculate healthy ageing score

| Items | Categorization |
| --- | --- |
| Ⅰ. Physical/ADL/IADL limitations (n=20) |  |
| Difficulty with jogging 1 Kilometers. | 0=some difficulty with the activity or could not do the activity; 1=did not have any problems with the activity. |
| Difficulty with walking 1 Kilometers. |  |
| Difficulty with walking 100 Meters. |  |
| Difficulty with controlling urination and defecation. |  |
| Difficulty with getting up from chair after sitting for long periods. |  |
| Difficulty with climbing several flights of stairs without resting. |  |
| Difficulty with stooping, kneeling, or crouching. |  |
| Difficulty with reaching or extending arms above shoulder level. |  |
| Difficulty with lifting or carrying weights over 10 Jin (5kg). |  |
| Difficulty with picking up a small coin from a table. |  |
| Difficulty with dressing. |  |
| Difficulty with bathing or showering. |  |
| Difficulty with eating. |  |
| Difficulty with getting in/out of bed and walking. |  |
| Difficulty with using the toilet, including getting up or down. |  |
| Difficulty with managing money. |  |
| Difficulty with taking medications. |  |
| Difficulty with shopping for groceries. |  |
| Difficulty with preparing a hot meal. |  |
| Difficulty with cleaning house. |  |
| Ⅱ. Cognition (n=7) |  |
| Cannot answer correct month given. | 0=can correctly answer; 1=cannot correctly answer |
| Cannot answer correct day of the month given. |  |
| Cannot answer correct year given. |  |
| Cannot answer correct day of week given. |  |
| Numeracy. |  |
| Immediate recall. | 0=could correctly recall at least 6 words; 1=could not correctly recall at least 6 words. |
| Delayed recall. | 0=could correctly recall at least 7 words; 1=could not correctly recall at least 7 words. |
| Ⅲ. Others (n=4) |  |
| Hearing problem. | 0=ever had;  1=never had. |
| Eyesight problem. |  |
| Speech problem. |  |
| Self-reported pain. | 0=a little/ somewhat/ quite a bit/ very often troubled with body pains;1=never troubled with any body pains. |
| Memory problem. | 0=ever diagnosed;  1=never diagnosed. |

**Table S2.** Variable description

| **Variable name** | **Definition or code** |
| --- | --- |
| Age | -- |
| Gender | 1=male; 2=female. |
| Residence | Residence indicates the household living region and is defined by National Bureau of Statistics of the People's Republic of China. 1=rural; 2=urban. |
| Education levels | Education level is a simplified version of 1997 International Standard Classification of Education (ISCED-97) codes. 1=Less than lower secondary education; 2=Upper secondary & vocational education; 3=Tertiary education. |
| Marital status | 1=married or partnered; 2=separated, divorced or widowed. |
| Household per capita consumption | Household per capita consumption is calculated by taking total  household consumption divided by the number of people in the household. The amount of total household consumption as aggregated from all consumption activities: food consumption in last week, non-food in the past 30 days, and other non-food consumption in the past year. The household per capita consumptions in different survey waves were adjusted by the Consumer Price Index, and then divided into four groups based on quartile. The details: (1)Low: <¥4018.75; (2)Low to middle: ≥¥4018.75 and <¥7061.76; (3)Middle: ≥¥7061.76 and <¥12326.66; (4)High: ≥¥12326.66. |
| Public health insurance coverage | 0 =the respondent is not covered by any public health insurance plan;1=the respondent is covered by at least one type of public health insurance plan, including Urban Employee Medical Insurance, Urban Resident Medical Insurance, New Cooperative Medical Insurance, Urban and Rural Resident Medical Insurance, Government Medical Insurance, Medical Aid or other government insurance plan. |
| Current work status | Current work status indicates whether the respondent engaged in any work in the past year. 0=the respondent is unemployed, retired, or never worked per the labor force status of each wave respectively;1=the respondent engaged in agricultural work, non-agricultural employed work, non-agricultural self-employment work, or non-agricultural family business work per the labor force status of each wave respectively. |
| Chronic condition | Chronic condition indicates whether the respondent ever suffered from chronic condition. 0=no, 1=yes, 2=morbidity. |
| Gave care to grandchildren | Gave care to grandchildren indicates whether respondent and spouse gave any informal care to their grandchildren in the past year. 0=the respondent and spouse did not provide any care to grandchildren in the past year;1=the respondent and spouse report looking after their grandchildren in the past year. |
| Live near children | Live near children indicates whether the respondent or spouse has a child who lives in the same city or county as the respondent. 0=they have living children but none of their children co-reside nor live in the same city or county as the respondent;1=any child co-resides or any non-co-resides child lives in the same city or county as the respondent. |
| Weekly contact with children | Weekly contact with children indicates whether a respondent or spouse has weekly contact with any of their children in person, by phone, by text message, by mail or e-mail. 0=the family respondent did not report any weekly contact;1=the family respondent reports any weekly contact either in person or by phone, mail or e-mail. |
| Gave money to children | Gave money to children indicates whether the respondent and spouse provided any economic assistance to their children or grandchildren in the past year. 0=the respondent and spouse did not provide any economic support to children or grandchildren;1=the respondent and spouse provided some economic support to children or grandchildren. |
| Received money from children | Received money from children indicates whether the respondent and spouse received any economic assistance from their children or grandchildren in the past year. 0=the respondent and spouse did not receive any economic support from children or grandchildren;1=the respondent and spouse receive some economic support from children or grandchildren. |
| Alcohol intake | Alcohol intake indicates whether the respondent has had an alcoholic beverage in the last 12 months. 0=the respondent reports not having any alcoholic beverage in the last 12 months;1=the respondent reports having had an alcoholic beverage in the last 12 months. |
| Smoking status | Smoking status indicates the respondent’s current smoking habit. 1=never smoke; 2=ever smoke but quit now; 3=still smoking now. |
| Social participation | Social participation indicates whether the respondent participates in any social activities in the past month. 0=the respondent does not participate in social groups or social activities;1=the respondent does participate in one of the social activities. |
| Physical examination | Physical examination indicates whether respondent does any physical examination within 2 years. 0=the respondent reported they do not do physical examination;1=the respondent reported they do physical examination within 2 years. |

**Table S3.** Bonferroni corrections of baseline descriptions

| **Variables** | **P value** |
| --- | --- |
| **Educational level** |  |
| “Less than lower secondary” vs “Upper secondary & vocational” | ＜0.001 |
| “Less than lower secondary” vs “Tertiary” | ＜0.001 |
| “Upper secondary & vocational” vs “Tertiary” | ＜0.001 |
| **Household per capita consumption group** |  |
| “Low” vs “Low-to-middle” | ＜0.001 |
| “Low” vs “Middle” | ＜0.001 |
| “Low” vs “High” | ＜0.001 |
| “Low-to-middle” vs “Middle” | ＜0.001 |
| “Low-to-middle” vs “High” | ＜0.001 |
| “Middle” vs “High” | ＜0.001 |
| **Chronic condition** |  |
| “None” vs “One” | 0.491 |
| “None” vs “Two or more” | 0.132 |
| “One” vs “Two or more” | 0.438 |
| **Smoking status** |  |
| “Never” vs “Quit now” | 0.997 |
| “Never” vs “Still” | ＜0.001 |
| “Quit now” vs “Still” | 0.026 |
